# A chance to stop and breathe: participants’ experiences in the North American Opiate Medication Initiative clinical trial

**DOI:** 10.1186/1940-0640-9-21

**Published:** 2014-09-29

**Authors:** Eugenia Oviedo-Joekes, Kirsten Marchand, Kurt Lock, Jill Chettiar, David C Marsh, Suzanne Brissette, Aslam H Anis, Martin T Schechter

**Affiliations:** 1 Centre for Health Evaluation & Outcome Sciences, Providence Health Care, St. Paul’s Hospital, 575- 1081 Burrard St., Vancouver, BC V6Z 1Y6, Canada; 2 School of Population and Public Health, University of British Columbia, 2206 East Mall, Vancouver, BC V6T 1Z3, Canada; 3 Centre Hospitalier de l’Université de Montréal, Hôpital Saint-Luc, CHUM Montréal, QC, Montréal H2X 3J4, Canada; 4 Centre for Addiction Research BC, University of Victoria, 2300 McKenzie Ave, Victoria, BC V8P 5C2, Canada; 5 Northern Ontario School of Medicine, 955 Oliver Road, Thunder Bay, ON P7B 5E1, Canada

**Keywords:** Opioid dependency, Diacetylmorphine, Injectable, Oral methadone, Opioid maintenance treatment, Qualitative methods

## Abstract

**Background:**

The North American Opiate Medication Initiative (NAOMI) clinical trial compared the effectiveness of injectable diacetylmorphine (DAM) or hydromorphone (HDM) to oral methadone maintenance treatment (MMT). This study aimed to determine participants’ perceptions of treatment delivered in NAOMI.

**Methods:**

A qualitative sub-study was conducted with 29 participants (12 female): 18 (62.1%) received injectable DAM or HDM and 11 (37.9%) received MMT. A phenomenological theoretical framework was used. Semi-structured interviews were audio-recorded and transcribed verbatim. A thematic analysis was used over successive phases and was driven by the semantic meanings of the data.

**Results:**

Participants receiving injectable medications suggested that the supervised delivery model was stringent but provided valuable stability to their lives. Females discussed the adjustment required for the clinical setting, while males focused on the challenging clinic schedule and its impact on employment abilities. Participants receiving MMT described disappointment with being randomized to this treatment; however, positive aspects, including the quick titration time and availability of auxiliary services, were also discussed.

**Conclusion:**

Treatment with injectable DAM (or HDM) is preferred by participants and considered effective in reducing the burden of opioid dependency. Engaging patients in research regarding their perceptions of treatment provides a comprehensive assessment of treatment needs and barriers.

**Clinical trial registration:**

NCT00175357

## Background

Opioid dependence is a chronic, relapsing disease that can be fatal if untreated [1]. Abstinence-based therapies, a first-line treatment choice in many countries, have been shown to successfully treat patients who have stable housing, family support, and personal motivation (e.g., readiness for change) [2]. However, the marginalization that often accompanies long-term opioid dependence [3] is associated with a decrease in the effectiveness of abstinence-based treatments, with less than 30 percent of patients remaining abstinent after one year [4]. Opioid substitution-based therapies, mainly oral methadone maintenance treatment (MMT), are considered the most effective options for the treatment of opioid dependence [2].

Although MMT is effective for many patients [5,6], it is estimated that 15–25 percent of the most severely affected individuals with opioid dependence are not reached or retained by MMT [7,8]. These individuals do not stay in MMT for very long, or they continue to use illicit opioids while in treatment [9,10]. Studies in Europe and Canada have demonstrated that supervised, medically prescribed heroin (i.e., diacetylmorphine, [DAM]) is an effective alternative for long-term opioid-dependent individuals not benefitting sufficiently from available treatments [11]. The clinical trial conducted in Canada—the North American Opiate Medication Initiative (NAOMI)—demonstrated the effectiveness of the supervised model of medically prescribed injectable DAM compared to oral methadone [12]. Participants randomized to injectable treatments in this study achieved a better clinical response and higher retention to treatment at 12 months, compared to those randomized to oral treatment [12].

Under this model, DAM is dispensed and self-administered by injection (or swallowed or inhaled in some clinics) under supervision in specially designed clinics. The rationale behind this model is that by providing pharmaceutical-grade heroin in the presence of health care providers, individuals not benefitting from other treatments are more likely to be attracted and retained in treatment. They are protected from common harms (e.g., fatal overdose and infectious disease transmission), since the dose and purity are controlled on site. This treatment also provides an opportunity to reduce illegal activities often arising from heroin addiction, by delivering medical and psychosocial support services as well.

Since the medication must be delivered on clinic premises (i.e., no ‘take-home’ doses), participants attend the clinic at least twice daily and undergo pre-intake (15 minutes) and post-intake assessments (30 minutes). Thus, providing injectable DAM might be a very demanding model for patients and could affect compliance. More generally, patient adherence to life-saving treatment for conditions such as diabetes, even when they can take the medications with them, has an average nonadherence rate of 24.8 percent [13]. A social researcher on addictions has criticized this aspect of the supervised model and suggested that qualitative research is needed to help understand how and why heroin prescription works so effectively [14]. These questions reinforce the need to include an evaluation of the intervention’s process and outcomes by the patients themselves to guide clinical decisions, as part of a patient-centered model of care [15-17].

Few studies have explored patient perceptions of injectable DAM treatment using structured interviews, satisfaction measures [18,19], and qualitative data [18-22]. A qualitative study with 21 participants and family taking part in the Andalusian clinical trial explored the perceived impact of medically prescribed DAM administered under supervision. The study described a change in participants’ perceptions and perceived significance of the substance (from *illicit drug* to a *medication)* and explored improvements in the workplace, family relations, and physical and mental health [20]. A study conducted in the Netherlands provided qualitative data from 24 participants who discussed the positive impact of having a reliable supply of DAM in improving their daily lives by reducing or discontinuing their involvement in illicit and street-based activities (e.g., drug dealing, hustling, and sex work) [21].

While we assessed satisfaction with injectable and oral treatments among NAOMI trial participants in a prior study [18], the questions regarding why the injectable group was more satisfied or why participants perceived the treatment to be effective remained unanswered. The aim of the present study was to further explore participants’ perceptions of the treatments delivered during the trial in order to improve our understanding of the effectiveness of these treatments and the model of care.

## Methods

### The NAOMI study

NAOMI was an open-label, phase III randomized controlled trial (RCT) that compared supervised injectable DAM and oral methadone in the treatment of long-term opioid dependence. The study was conducted in Vancouver and Montreal, Canada, between 2005 and 2008. Participants were aged 25 or older, with at least 5 years of opioid dependence, current daily injection of illicit opioids, a minimum of two previous treatments for opioid dependence, including at least one opioid substitution treatment (OST) attempt, and no enrollment in OST within the prior 6 months. A full description and discussion of the participants’ profiles, study design, methodology, and main results have been published elsewhere [12,23,24]. Briefly, a total of 251 individuals were randomized to receive oral methadone (n = 111), injectable DAM (n = 115), or injectable hydromorphone (HDM) (n = 25); the latter two on a double-blind basis. Participants administered the injectable medications up to three times daily under the supervision of clinic staff, and oral methadone was dispensed daily. Trial treatments were provided for 12 months, with an additional 3-month period to taper and transition to other treatment modalities (primarily methadone). Participants met with their study physician at least once per month for reviewing and discussing their medication. Participants had access on site to a comprehensive range of psychosocial and other primary care services, including referral to specialists and treatment for concurrent disorders. The procedures followed in the trial were in accordance with the Helsinki declaration [25].

### NAOMI Participants’ experiences study

#### Study design

The NAOMI participants’ experiences study was a qualitative sub-study that aimed to understand the impact of the NAOMI trial on participants’ lives. A phenomenological theoretical framework was chosen [26]. This study design was most appropriate for providing a description of the meaning and significance of participants’ prior lifetime experiences and appraisals of the treatments received during the clinical trial [26,27]. Participants eligible for this qualitative sub-study were beyond the 12-month NAOMI treatment period (to avoid interference with the RCT’s primary outcome measures at the Vancouver site).

Participants were selected through stratified probability-based sampling. Stratum included male and female gender, Aboriginal and non-Aboriginal ethnicity, and oral and injectable treatment groups. Although less commonly used in qualitative research [28], the stratified probability-based sampling was most appropriate to ensure that a nonbiased and representative sample of participants was included, improving credibility [29]. A total of 32 participants were randomly selected, representing the stratum defined above. This sample size was directed by the sub-study’s research questions and goal for representativeness.

#### Data collection procedure

The University of British Columbia/Providence Health Care Research Ethics Board approved the study, and all participants provided written informed consent. Semi-structured qualitative interviews were conducted by research staff, independent of the clinical team, between February and September 2008. The study coordinator (JC) invited participants and explained the aims of the qualitative sub-study. Participants received monetary compensation for their time.

Interviews were held one time only with consenting participants at the NAOMI research office, with either the parent study coordinator (JC) or the second interviewer (parent study research staff). Both interviewers were female and were experienced with the study population in general. By the start of this qualitative study, the interviewers had met with participants several times before (every 3 months) to conduct follow-up research interviews for the parent trial that had started 3 years prior. Therefore, the interviewers had a contextual grasp of the participants’ lives, and participants were familiar with them. The research interviewers for the sub-study received additional training on conducting semistructured interviews as well as collecting data on the topics included in the topic guide.

A semistructured topic guide was used with an open-ended questioning approach. Topics included: drug use history and prior addiction treatment, situation before NAOMI, expectations of NAOMI, situation during NAOMI, situation after NAOMI, and general perceptions of the NAOMI experience. When necessary, interviewers repeated or rephrased questions to ensure that the topics were covered; prompting was not used. Interviews lasted between 45 minutes and 2 hours. All interviews were audio-recorded and transcribed verbatim by an independent contractor.

The parent study research coordinator and study investigators (EOJ, MTS) met regularly during the data collection phase to review and discuss the data collected. Upon completing 29 interviews, it was agreed by the team that no new information was being gathered from the interviews and across each stratum.

#### Data analysis

An inductive thematic analysis approach was used. The thematic framework established by the researchers (EOJ, KM, DCM, MTS) was semantic and driven by the explicit meanings of the data [30]. This framework was organized according to the main topics of the semistructured interviews. Data analysis was led by two of the authors (EOJ, KM) over successive phases. All coding and analysis was done using NVivo software for qualitative data analysis [31].

In the first phase, prior to any coding taking place, EOJ and KM first read each transcript to familiarize themselves with the data. During the second phase, initial ‘free’ codes were created based on semantic content. The lead researchers reviewed the initial free codes together and created the following major themes during the third phase: a) reasons for participating in NAOMI; b) perceived impact of NAOMI treatment; c) perceptions of the delivery of NAOMI treatment; d) recommendations; and e) experiences with ending NAOMI. Further review and refinement of the data was done in the next phase of analysis. The content of the major themes was reviewed; minor themes were identified based on predominating patterns [32].

After this phase, the researchers met to review preliminary thematic maps to refine and define themes for the objectives of the present paper. During the analysis, comments were compared by gender (male and female), ethnicity (Aboriginal and non-Aboriginal), and treatment characteristics (treatment arm, retention, and response). These characteristics were also used to identify participants’ quotes.

## Results

Participants’ characteristics were representative of the parent clinical trial (Vancouver) sample [23]: 12 (41.4%) female and 17 (58.6%) male, Aboriginal ethnicity (27.6%), and average age of 44. A total of 14 participants (48.3%) received DAM, 4 (13.8%) received HDM, and 11 (37.9%) received oral MMT. Nineteen (65.5%) participants were considered responders, and 25 (86.2%) were retained in treatment.

### Reasons for participating in NAOMI

Participants in both treatment arms entered NAOMI with the hope that they would be randomized to injectable treatments. Their reasons for preferring the injectable treatment varied, but were rooted in their prior experiences with other addiction treatments, mainly oral methadone. Some participants described wishing to be part of NAOMI to obtain “free heroin,” in the context of not having to hustle for a period of time. Others expected that NAOMI would alleviate financial distress, provide stability, reduce involvement in sex work, and assist them with improving their health and social situation.

*It wasn’t about gettin’ high for me. It was about getting a life… That’s why I had went in…It was about not being sick. And, taking away that obsession…I wish I was still in it. I do.* [female non-Aboriginal participant, age 50, DAM, nonresponder, retained]

*I could not manage, my pain, uh, when I first heard about it [NAOMI]. I was, I was in, let’s say, [sigh] medium, or, or, mild chronic pain. If, if, I did have a round-the-clock pain, which happened quite often, it would uh be kind of low level and could be treated with drugs. But I just, [pause] I have a hard time staying on methadone. It doesn’t seem to do anything for me and it doesn’t, doesn’t help the pain. Doesn’t, so uh, yeah, so that’s, that, I’ve tried it a number of times. And it just doesn’t seem to work for me. So I was hoping that I would get randomized to be heroin…. And of course I didn’t.* [male non-Aboriginal participant, age 40, MMT, nonresponder, not retained]

### Perceived impact of NAOMI treatment

#### Illicit drug use

Participants in both treatment arms (mainly injectable participants) described a significant reduction in illicit heroin use during NAOMI. Participants reflected that the reduction in heroin use was due to the quality of the medication and the consistency of the dose received in the clinic.

*But I didn’t have any, uh physical need to, to do heroin, outside of the programme. I didn’t, there was enough dosage for me to uh, as long as I made the sessions, I didn’t need.* [male non-Aboriginal participant, age 50, DAM, responder, retained]

Some of these participants, including one oral participant, also said that they stopped using street heroin for the entire time they were receiving study treatments. Those receiving injectable treatment expressed their lack of physical need or a desire to engage in ‘the hustle’ to obtain illicit heroin.

*I didn’t have to steal. I didn’t have to rob people. I didn’t have to do things like that. My drugs were taken care of.* [male non-Aboriginal participant, age 40, HDM, responder, retained]

Few described occasional use of street heroin, mostly due to continued use of ‘speedballs’ (i.e., injection of cocaine and heroin) or simply due to curiosity.

*I smoked rock* [crack cocaine] *every day. And, but uh, heroin I did it, I bet you I could count on one hand the amount, the times I did heroin during the whole NAOMI thing.* [female Aboriginal participant, age 50, DAM, responder, retained]

In addition to heroin, crack cocaine was the most common illicit substance discussed by participants. During NAOMI, the majority of participants (mostly those receiving injectable treatment) indicated experiencing either a decrease or no change to their crack cocaine use. Reasons for reducing crack cocaine use varied; many participants stated they were *‘tired, sick of it, realized what life was like before they started doing drugs;’* one participant (DAM female, nonresponder, retained) stated that she reduced crack cocaine use because she was not engaging in sex work as often as before NAOMI.

A few participants reported an increase in crack cocaine, and their discussions about this were more elaborate than those who described no change or a reduction in crack cocaine use. This increase was described by male participants only, regardless of randomization arm, response, or retention to NAOMI treatment. Participants associated their increased crack cocaine use with having more freed-up funds and the ‘*engrained*’ lifestyle associated with daily drug use. Other aspects of participants lives, such as the environment in which participants lived, their relationships, and their sources of income (i.e., drug dealing), were described as challenges to completely stopping illicit drug use.

Participant: And I just, you know, the financial, uh, freedom from having to buy heroin every day. Like you known more money in my pocket and, unfortunately, rather than spend it um more um, sane fashion. I got to uh, a lot of it went to uh, crack and cocaine.

Interviewer: Right. So, do you think? How, how could that be any different? Really, how, could NAOMI have accommodated that or other treatment programmes in the future?

*Participant: Um, it’s not easy. If it wasn’t in the neighbourhood, you know.* [male non-Aboriginal participant, age 50, DAM, responder, retained]

#### Illicit activities

Prior to NAOMI treatment, participants discussed their histories of engaging in drug dealing, petty theft, and sex work (female participants only) as a means of supporting illicit drug use. Only those receiving injectable treatment indicated no longer engaging in these activities, given the provision of treatments in NAOMI.

*I didn’t have to steal. I didn’t have to rob people. I didn’t have to do things like that. My drugs were taken care of.* [male non-Aboriginal participant, age 40, HDM, responder, retained]

*It got me out of the sex trade. The survival sex trade.* [female non-Aboriginal participant, age 50, DAM, nonresponder, retained]

#### Housing

Housing situations varied considerably during the study. Some participants said they were living in the same place for years, others (mostly females) acquired housing during NAOMI with the help of study staff, and two were homeless at the time of the interview (both nonresponders). Participants described how difficult it was to acquire a secure place to live, expressing that they could not afford decent places and needed to accept poor conditions in order to have a place to live. Participants perceived their drug use as a barrier to housing and believed lack of housing was a barrier to work. Participants also were not welcome at family or friends’ homes. Most of those who were homeless for any period of time in their life indicated that when they were clean, they found a place to live (although the conditions were not always very good due to the lack of affordable and liveable places).

*I was only homeless, for I don’t know, 3 months or something like that. I mean technically I’m homeless right now, but I, I haven’t actually wound up on the streets, you know. I got places to stay. And mean that’s the thing. So when I went on the methadone, the need for money isn’t as desperate. So um, I’m a little more tolerable to be around, like I’m welcome at friend’s houses and, to be at my dad’s house and stuff, because I’m, you know. [pause] They’re not worried that I’m all of a sudden start, [pause] start directly into, into opiate withdrawal. And, crying, begging, for money. [chuckle] kind of like that, right? so. It’s more relaxing to be around me I suppose.* [male non-Aboriginal participant, age 40, MMT, responder, retained]

#### Health

At initiation to NAOMI, participants described having both physical (i.e., infections, respiratory and dental problems) and mental health conditions (i.e., anxiety, depression, and suicidal ideation). Participants also described suffering from chronic physical pain (e.g., arthritis, headaches, and injury-related pain) and emphasized that this was one of the reasons for using illicit opioids and for seeking NAOMI treatment. Regardless of treatment arm, response, and retention, participants experiencing mental and physical health problems discussed how NAOMI’s health care staff provided prescriptions for bipolar disorder and antiretroviral medications, as well as referrals for acute and chronic conditions, such as arthritis, infections, and skin conditions.

*And my health got pretty good too…Well, I’m HIV so, my, uh, viral load went down. They [NAOMI doctors] were [inaudible] I guess, yeah. I like ‘em. Well, some of the time I went in there with injuries, so, so they took care of that while I was there, you know? They knew about addiction I guess, so, that was handy, yeah.* [male Aboriginal participant, age 40, DAM, responder, retained]

### Overall impact of NAOMI

Overall, participants described a positive impact of the study on their lives. There were only two negative comments about the impact of NAOMI: randomization for those assigned to oral and ending the study treatment for those receiving injectable (described below in the Ending NAOMI section). Participants in the oral group articulated their disappointment with the outcome of the randomization using expressions such as *“disappointed, devastated, bitter, went violent, wasn’t excited, like a big bomb had dropped, I cried.”* Some decided to give oral methadone another try right away; others left and eventually returned to treatment days or weeks later.

*I came back about a month later and decided to go on the methadone. […] Yeah the whole world just fell out from beneath me when they said the word methadone.* [male, non-Aboriginal participant, age 40, MMT, responder, retained]

Participants in both treatment arms (including nonresponders) indicated that the treatments provided them with stability, improved their sense of self-worth, strengthened their personal and community relationships, and allowed them to reflect positively on their future.

*And what the NAOMI project did for me was…let me realize what my life was before I started doing drugs and had to spend all my money on drugs and all my free time, on getting money for drugs. And actually made me realize like the other parts of my life, that I had before, that I basically forgot about in the many years that I was doing heroin. And right now, to this day, I don’t do, any injection drugs at all…I feel a lot better about myself too, like I have self-worth again. And when I was an addict, doin’ dope, fuck I just, felt dismal about myself.* [male non-Aboriginal participant, age 40, DAM, responder, retained]

### Perceptions of treatment in NAOMI

#### Interactions with health care providers

A common theme among participants in both treatment arms was their interactions with health care staff at the study clinic. Few participants described a negative interaction with health care staff. Participants were more detailed during discussions about their interactions with the physicians, but spoke positively about their general interactions with health care staff. They described receiving care that was *respectful, nonjudgmental, honest,* and felt the staff *listened, really cared, understood, and were always there to answer a question.* The staff’s experience and familiarity with the participants was beneficial because staff were able to be proactive on addressing the various needs of participants. For example, participants discussed how the social workers helped address housing and disability support needs.

When making comparisons between the quality of care received at the study clinic with prior experiences in community-based centers, participants in both treatment arms emphasized their preference for the care at NAOMI clinics because the physicians were more aware of their needs, and they could be more open about expressing these needs.

*Uh actually yeah he referred me to um to um what do you call this? Um bone specialist…What do you call this? Uh rheumatologist. He hooked me up with my rheumatologist. Um at one point I had skin problem. He got me an appointment with a skin specialist. I mean he takes good care of me. And he explained to me everything. He’ll turn his computer screen if I don’t understand something and explain it to me. He, he’s very good. I never, I never ran into a doctor like that before in my life.* [male non-Aboriginal participant, age 40, MMT, nonresponder, retained]

#### Methadone maintenance treatment

Participants receiving oral treatment indicated they preferred the delivery of MMT at NAOMI clinics because the services and staff were more easily accessible (i.e., no wait times, pharmacy on site). A few participants also expressed their preference for NAOMI’s titration protocol, stating that the physicians increased doses in a manner that was appropriate for the participant, allowing them to reach a more stable and comfortable dose quickly. Participants suggested that being able to reach more satisfactory doses and having a role in this decision was critical at the beginning of treatment, as it reduced withdrawal symptoms and illicit heroin use.

*I think the main thing is…Dr. XX put me up to, high enough dose. Where I said before, I think I was on 80 before when I was at this other clinic. But I mean it wasn’t, it wasn’t enough. By the time I came to, it’s quite a few hours before it came time to get my dose for the next day. I was starting to, go through withdrawals. And I was on methadone for like 2 months on that dose and I was using all the time. And the doctor wouldn’t put me up any more.* [male, non-Aboriginal participant, age 40, MMT, responder, retained]

In light of these positive experiences, the main recommendation for improving oral treatment in NAOMI was to extend the operating hours of the clinic.

#### Injectable treatments

Common topics about the logistics of treatment delivery included lengthy pre- and post-assessment times, short operating hours and lack of flexibility to accommodate work schedules, and the overall amount of time spent at the clinic. Males discussed the schedule, work, transportation, and rules of the clinic.

*And uh, I found it to uh, going three times a day was almost, a full-time job…Cause I mean, three times, each, each session you know? With travel time there and back it, it took you know, like, you know, between five and six hours a, a, day, right? And it’s basically impossible to uh, try and go back to work.* [male non-Aboriginal participant, age 50, DAM, responder, retained]

Some participants, mostly female, discussed the adjustment that was required for injecting in a supervised setting, including changes to the routine (i.e., preparation of own drug) and injection site (i.e., jugular, leg). Another complaint that a few participants discussed was the time restriction set for the injection room (approximately 7 min). The reason for their difficulty with self-administering the injectable medications was because of poor vein health resulting from many years of injection drug use.

*It was hard for me because I’m not used to being around people when I did mine, right. And it made me nervous so I always had a hard time.* [female Aboriginal participant, age 50, HDM, responder, retained]

Participant: Well, difficult, cause, um, being uh, shooting dope for so long, and I don’t have any veins left, so, the timeframe, and, uh, to hit being so large uh, and in turkey baster syringe. and having a hard time getting a vein, let alone in 7 minutes. And, I ended up trying to, to do it intramuscularly, uh a lot of times. [inaudible]

Interviewer: Uh huh [affirmative] and did that change your high then?

*Participant: No. it’s uh, there was enough of it, in uh, you know, just, just takes a couple minutes and, to kick in.* [male non-Aboriginal participant, age 50, DAM, responder, retained]

Participants also described the dose and quality of the medications they were receiving in NAOMI as being significantly better compared to street heroin. The improved drug quality allowed them to worry less about what other unknown substances they were inadvertently taking.

*Now, my first shot in NAOMI was 15 milligrams. The effect I got off of that was damned close to the effect I got off of shooting a quarter gram [of street heroin]…that’s very drastic. And uh, it’s an eye opener, man…that probably alone would have given me, reason enough to, consider treatment of some kind.* [male non-Aboriginal participant, age 40, DAM, responder, retained]

### Recommendations for improving the delivery of injectable treatment

Logistics of the treatment were the most commonly referenced recommendation for improving NAOMI. Participants suggested changes to the structure of the program, including longer operating hours, more flexibility in session times, and less time required for each visit. A few male participants discussed the interference of the strict schedule on their ability to work. Additional programs were also a common recommendation, including vocational and life skills support, as well as treatment for other drug use. Although the treatment brought stability to participants’ lives, there were concerns about how best to use the time freed by the treatment.

*If this was to ever get off the ground and be a permanent thing, that something be implemented…to take up this free time…maybe train for this or go to school part-time or something just so you don’t have all that time on your hands.* [male Aboriginal participant, age 51, DAM, responder, retained]

Participants also suggested that the treatments should have been provided for a longer period of time, or transitioned into a program, in order to enhance and sustain the benefits of the treatments.

*Because by the time it was over that was when I really felt that I was starting to stabilize, you know… on my lifestyle… But if I’d had more time, maybe I would have been able to…go another couple of routes, like, uh, think about school or maybe even a job. But…it was over a little too quick for that.* [male Aboriginal participant, age 40, DAM, responder, retained]

### Ending NAOMI

Participants receiving injectable treatment primarily discussed the process and emotions of ending NAOMI. Many study participants reported that they were able to transition or continue on MMT with their NAOMI study physician, either at the site of NAOMI or in community-based clinics. A few refused MMT because it did not work for them or because of unsuccessful transitions (e.g., disagreements with health care provider). Some participants receiving injectable treatments discussed how NAOMI enabled them to consider engaging in other treatment options (i.e., detox, MMT) that previously would not have been considered.

*Well, you know one of the effects of NAOMI and I’m not sure how or why, I am more accepting of methadone now than I was before. More able to, uh you know, to accept, the reality of it. You know, uh [pause] I’m not using as much opiates as I did before, uh, at NAOMI, you know? And I, I don’t, run to find opiates as quickly as I used to.* [male non-Aboriginal participant, age 51, DAM, responder, retained]

Participants indicated that even though they understood that injectable treatments were not guaranteed beyond the end of the study, they experienced anxiety, fear, sadness, and stress due to ending the study.

*And I was never under any illusions. And you know, again, on the street people would say, yeah, but what are you gonna do when it’s over? What I would do the same as I did before. When it’s over. You know, it’s a respite. It’s a gift. And I’m not gonna sit here and complain about it.* [male non-Aboriginal participant, age 51, DAM, responder, retained]

Participants remained hopeful that the evidence from NAOMI would expand treatment options in Canada, and they were happy to have been part of the study. None of the interviewed participants expressed regret at being part of NAOMI.

*I was happy to be part of it…it gave me a bit of hope into the future of um, addictions treatment in […] Canada. Um, [pause]…there was an adjustment period, when I got out of NAOMI there. I was sad. And I was depressed for a little while, because you know, when you have something like that comes to an end, not only did I really miss the comfort and the attention and the really caring staff, which I do still miss.* [male non-Aboriginal participant, age 40, DAM, responder, retained]

## Discussion

The aim of this study was to explore how participants in the Canadian clinical trial testing injectable opioids perceived the treatment received. The participants’ perspectives of treatment with medically prescribed DAM were concordant with the rationale of this model of care: by providing pharmaceutical-grade heroin in a supervised health care setting that also provided comprehensive health services, we engaged street heroin-injecting individuals to treatment and offered them an opportunity to reduce harms and illegal activities and improve their health.

A prior study showed that participants in NAOMI applied to the clinical trial mainly to receive “free heroin” [33]. In this study, the participants signified the possibility of receiving free heroin as an opportunity for getting their lives back. The provision of medically prescribed injectable opioids is described as introducing stability in their lives. Now that the substance they were dependent on was taken care of, there was no need to be involved in illicit activities or sex work and they could direct their resources to finding housing and possibly employment. Also, through the daily contact with health care workers, they were diagnosed and treated for other comorbidities.

This study provides an explanation as to why this treatment is so effective, despite the strict regimen of the supervised delivery model [14]. While participants discussed the challenges associated with the logistics of treatment delivery (e.g., schedules, no take-homes, injecting among strangers), it does not reflect the emotions and intensity in the way they described their lives while they were untreated: waking up in a panic, stealing, feeling hopeless, depressed, and like a “*total wreck.”* The discourses recorded in this study suggest that participants found the supervised delivery model stringent, but they highly valued the stability it brought to their lives. This stability is described mostly in relation to the provision of the injectable medications they considered beneficial, but also in the supportive model of care. These results are consistent with the findings of the Dutch and Andalusian reports on participants’ perceptions of this treatment [20,21]. For example, in the Dutch study, participants complained about how the tight clinic schedules interfered with their daily activities, such as household activities or work. However, this schedule also provided structure to their lives, mostly among those engaged in street activities, such as sex work or drug dealing [21].

This study shows that the treatment provided was considered effective at stopping the use of illicit opioids, confirming our prior results [12]. As expected, participants receiving injectable opioids at the clinic reported not using street opioids. As in prior studies [19-21], our data show that the secure drug supply translated into a significant decrease of engagement in illicit activities and the daily street hustle. This release of time, and in some cases resources, introduces an important challenge for patients and the community: how to fill this free time. For example, participants in the Andalusian study conveyed the challenge of suddenly not having to spend the day going after heroin and how their needs beyond heroin became evident, such as lack of skills to obtain employment [22]. Results from the parent trial found no changes in the use of cocaine from baseline [12], and accordingly, in this study most of the participants reported having reduced or not changed their cocaine use. However, those few whose cocaine use increased attributed it to having more funds available and their struggle to disengage from the drug scene.

In NAOMI’s analysis of satisfaction with treatment, after controlling for treatment effectiveness, those randomized to the injection group were significantly more satisfied with treatment than those in the MMT group [18]. From the open-ended comments section of the Client Satisfaction Questionnaire, participants in both arms of the study described the need for additional ancillary and nutritional services. The present findings confirm this, with participants expressing that vocational and skills training would be beneficial programs as participants begin to experience stability in their lives and are confronted with the need to plan for their future.

The gender-based analysis in the NAOMI study found that retention and clinical response to DAM treatment were slightly lower among women than men (though not statistically significant) [34]. In the qualitative analysis, men and women had similar discourses, except women discussed the adjustment required for the environment and physical site of injectable treatment administration (e.g., the public environment of the injection room), whereas males focused on the challenging schedule of the treatment and its impact on their ability to work. Women’s experiences with the logistics of the clinical care setting may explain the slightly reduced retention and response rates.

Some participants (mainly male) were concerned about the schedule of the treatment and its impact on their ability to work. The potential impact on the capacity for employment of coming to a clinic two to three times per day has been raised as an issue of this treatment model [35]. However, there are many alternatives under this model that would facilitate patients adjusting to a work schedule. For example, clinics in Europe offer a combination of injectable and oral long-acting opioids (e.g., methadone), and have diversified options (e.g., methadone, morphine, DAM, etc.) for managing individual patient needs [36]. Also, several studies show that after a period of stabilization some patients voluntarily transition to oral methadone or abstinence-oriented treatment [37,38], options that allow a schedule more amenable to work.

Due to clinic capacity, participants entered the trial on a staggered schedule to complete the planned sample size over time. The last participants finished the treatment in April and June 2008, respectively, in Vancouver and Montreal. This study collected data between February and September 2008. Participants expressed their absolute disappointment with the end of the injectable treatments and explained the negative impact on their lives. Regardless, they saw the NAOMI experience as a positive one. They referenced mostly two aspects: First, for a period of time, they obtained stability and had the chance to make plans beyond how to obtain the drug. Second, they had the hope that NAOMI would become a program and were proud of having helped to build evidence for this case.

This study provides further explanation of why treatment with injectable DAM, despite the supervised model of care, can be effective. Our evidence adds to the current (and limited) data on participant perceptions of treatment with injectable DAM for long-term opioid dependence. Such data are particularly useful as future programs with this treatment are developed. Patient engagement in chronic disease treatment is essential to promoting treatment access and retention, thus reducing the burden of disease for the individual and society.

## Competing interests

The authors declare that they have no competing interests.

## Authors’ contributions

EOJ and KM made substantial contributions to analysis and interpretation; EOJ also led preparation of the manuscript. KL and JC made substantial contributions to the acquisition of NAOMI data and assisted with drafting the manuscript; DCM, SB, AA, and MTS made substantial contributions to NAOMI’s conception and design and interpretation of the present data. All authors read and approved the final manuscript and agree to be accountable to the integrity of the work.
